# Analysis of Vehicle-Following Heterogeneity Using Self-Organizing Feature Maps

**DOI:** 10.1155/2014/561036

**Published:** 2014-11-05

**Authors:** Jie Yang, Ruey Long Cheu, Xiucheng Guo, Alicia Romo

**Affiliations:** ^1^Development Research Center of Transportation Governed by Law, Southeast University, Nanjing, China; ^2^Department of Civil Engineering, The University of Texas at El Paso, El Paso, TX 79968, USA; ^3^School of Transportation, Southeast University, Nanjing, China; ^4^Turner-Fairbanks Highway Research Center, Federal Highway Administration, McLean, VA 22101, USA

## Abstract

A self-organizing feature map (SOM) was used to represent vehicle-following and to analyze the heterogeneities in vehicle-following behavior. The SOM was constructed in such a way that the prototype vectors represented vehicle-following stimuli (the follower's velocity, relative velocity, and gap) while the output signals represented the response (the follower's acceleration). Vehicle trajectories collected at a northbound segment of Interstate 80 Freeway at Emeryville, CA, were used to train the SOM. The trajectory information of two selected pairs of passenger cars was then fed into the trained SOM to identify similar stimuli experienced by the followers. The observed responses, when the stimuli were classified by the SOM into the same category, were compared to discover the interdriver heterogeneity. The acceleration profile of another passenger car was analyzed in the same fashion to observe the interdriver heterogeneity. The distribution of responses derived from data sets of car-following-car and car-following-truck, respectively, was compared to ascertain inter-vehicle-type heterogeneity.

## 1. Introduction

Vehicle-following has been an important topic of traffic flow research in the past 50 years. Many deterministic vehicle-following models have been proposed and studied [1] and many of them are being used in microscopic traffic simulation tools [2]. Earlier studies, for example [3], relied on limited sets of data collected from instrumented vehicles driven in test tracks. Results of the earlier studies have been developed into the well-known Gazis, Herman, and Rothery or simply the GHR model [3, 4]. Users of the GHR model or other deterministic models have assumed that the selected model, once calibrated with its fixed parameter values, was applicable to all driver-vehicles; that is, the driver-vehicle population is homogenous. Some microscopic traffic simulation tools distinguish the behavior between different driver-vehicles by using the same model but vary the parameter values between different driver-vehicles.

With the large-scale vehicle trajectory data collection efforts enabled by remote sensing techniques in the past decade, several researchers have begun studies on heterogeneous vehicle-following behavior between driver-vehicles and/or for the same driver-vehicle [5–8]. Such studies still relied on one or more prespecified vehicle-following equations. The researchers either (i) calibrated different equations to show that different driver-vehicles responded with different driving rules; (ii) calibrated the same equation but different parameter values between driver-vehicles; or (iii) calibrated the same equation but different parameter values between acceleration and deceleration. Such studies still depend on the deterministic equations, which may need to be calibrated to different segments of the driver-vehicle population.

In this paper, we use the term vehicle-following instead of the conventional term car-following, as the lead or following vehicle may be a truck instead of a car. We propose to use the self-organizing feature map (SOM) to replicate vehicle-following behavior. The SOM consists of neurons arranged systematically on a two-dimensional surface (known as a “map”). Each neuron has a prototype weight vector that represents the characteristic features in the input space. Such structure is capable of mapping patterns in the high dimensional input space into a two-dimensional map. According to the unsupervised learning rule, vectors that are similar to each other in the multidimensional space will be clustered in the same neighborhood in the SOM's two-dimensional space, which makes it possible to be adopted as a tool of data classification. Conventional neural networks do not have the unsupervised clustering capability. Because of its unique structure, users of the SOM do not need to specify the function between the input features and its output variable. No equation needs to be predefined and no parameter calibration is necessary. Therefore, the SOM may be considered as a nonparametric approach to model vehicle-following.

The objective of this paper is to use the SOM to study the heterogeneities of vehicle-following behavior. We use a trained SOM to show that when presented with similar stimuli (i) different car drivers respond with different magnitudes of acceleration when following cars; that is, car drivers have interdriver heterogeneity; (ii) the same car driver responds with different magnitudes of acceleration when following the same car; that is, the same driver has intradriver heterogeneity; and (iii) car drivers respond with different magnitudes of acceleration when the leaders are of different vehicle types. We called this phenomenon inter-vehicle-type heterogeneity. In additional to proposing the SOM as a nonparametric vehicle-following model, the findings of interdriver heterogeneity, intradriver heterogeneity, and inter-vehicle-type heterogeneity serve as complements to limited earlier studies.

After this introduction, the next section of this paper reviews the vehicle-following models and SOM. This is followed by a description of the data used in this research. The next section presents the SOM training. Subsequently, we present the results of using the trained SOM to analyze the interdriver, intradriver and inter-vehicle-type heterogeneities. The findings are summarized towards the end of this paper.

## 2. Literature Review

### 2.1. Vehicle-Following Models

A vehicle-following model is an equation (or a set of equations) that describes the movement of a driver-vehicle in response to the dynamics of the driver-vehicle immediately ahead, when both vehicles are traveling in the same direction in the same lane. As a fundamental building block of microscopic traffic simulation, the realism of a vehicle-following model improves the accuracy of the simulation outcome, which in turn enables better transportation decision making.

The historical development of vehicle-following models from 1958 to 1999 has been summarized in [1]. Many vehicle-following models have been proposed, tested, and used in microscopic simulation models over the years [2]. The deterministic model proposed by Gazis, Herman, and Rothery [3], often known simply as the GHR model, is one of the earliest and the most well-known models. The GHR model, also known as the General Motors (GM) model, takes the following form:

(1) $$\begin{array}{c} {\ddot{x}}_{f}\left(t+\Delta t\right)=\lambda_{f}\frac{{\left[{\dot{x}}_{f}\left(t+\Delta t\right)\right]}^{m}\left[{\dot{x}}_{l}\left(t\right)-{\dot{x}}_{f}\left(t\right)\right]}{{\left[x_{l}\left(t\right)-x_{f}\left(t\right)\right]}^{k}}, \end{array}$$

where ${\ddot{x}}_{f}\left(t\right)$ is the acceleration of the follower *f* at time *t*; ${\dot{x}}_{f}\left(t\right)$ is the velocity of the follower *f* at time *t*; ${\dot{x}}_{l}\left(t\right)$ is the velocity of the leader *l* at time *t*; *x*_*f*_(*t*) is the position of the follower *f* at time *t*; *x*_*l*_(*t*) is the position of the leader *l* at time *t*; *λ*_*f*_ is the follower's sensitivity constant; Δ*t* is the time lag in the follower's response; and *m* and *k* are calibration constants.

The GHR model equates the follower's response to the follower's sensitivity multiplied by the external stimulus. The calibrated *m* and *k* values obtained in different studies have been summarized in [1]. The different *m* and *k* values found at different study sites gave an indication of the heterogeneity of vehicle-following behavior across locations. Among other vehicle-following models that have been studied extensively are the Helly model [9], Gipps model [10], and Intelligent Driver model [11]. Although these models take different functional forms, they share the same characteristics of having the follower's acceleration ${\ddot{x}}_{f}\left(t+\Delta t\right)$ as the response, and follower's velocity ${\dot{x}}_{f}\left(t\right)$, relative velocity $\left\lfloor {\dot{x}}_{l}\left(t\right)-{\dot{x}}_{f}\left(t\right)\right\rfloor$, and space headway ⌊*x*_*l*_(*t*) − *x*_*f*_(*t*)⌋ among the stimulus terms.

Earlier vehicle-following studies have assumed that the model form and constants, once calibrated, applied to all the driver-vehicles or at least all passenger cars observed at the same site. Most of the available traffic simulation models, such as CORSIM [12] and VISSIM [13], assume one model form for all the driver-vehicles but account for variation between driver-vehicles by assigning different parameter values. In CORSIM, there are 10 types of drivers; each represents a different degree of aggressiveness in vehicle-following. Each vehicle generated in a CORSIM model is randomly assigned one type of driver. In VISSIM, users are able to define the probability distributions of desired speed, maximum acceleration, and other vehicle performance parameters.

Recently, researchers have begun to study the different responses between drivers (interdriver heterogeneity) and for the same driver (intradriver heterogeneity, part of it is also known as asymmetric behavior) when presented with similar stimuli. Brockfeld et al. [14] and Ranjitkar et al. used trajectory data collected from nine vehicles driven in a test track in Hokkaido, Japan, using Global Positioning System receivers to calibrate many vehicle-following models [15]. They found that different vehicle-following models produced different error magnitudes after parameter calibration. They noted that the variation of errors between drivers were larger than the variations between different vehicle-following models. Ossen and Hoogendoorn fitted the parameters *λ*_*f*_, *m*, and *k* of the GHR model to a vehicle trajectory data set collected at the A2 Motorway in Utrecht, the Netherlands [16]. They found that different drivers had different calibrated *λ*_*f*_, *m*, and *k* values. Punzo and Simonelli fitted four vehicle-following models to vehicle trajectory data collected in Naples, Italy [17]. They found a high degree of variability of the calibrated parameter values among drivers and also for the same drivers under different driving conditions. This is perhaps the first report on the observation of intradriver heterogeneity. Ossen et al. again attributed the difference in the observed vehicle-following behavior between drivers to (i) different vehicle-following equations and (ii) different parameter values of the equations [18]. Kesting and Treiber calibrated the Intelligent Driver model and the Velocity Difference model to a vehicle trajectory data set [19]. They found that intra-driver variability rather than interdriver variability accounts for a large part of the calibration errors.

Siuhi and Kaseko appear to be the first to use the Next Generation SIMulation (NGSIM) vehicle trajectory data set to analyze vehicle-following behavior [6]. They calibrated the GHR model (without the Δ*t* term in the follower's velocity) using the data collected at the U.S. 101 Freeway in Los Angeles. They showed the distributions of Δ*t* during acceleration and deceleration, with deceleration having a smaller mean Δ*t* value. The same study also analyzed the distributions of *m* and *k* values and recommended different sets of *m* and *k* values for acceleration and deceleration, respectively, even for the same drivers. The different Δ*t*, *m*, and *k* values in acceleration and deceleration lead to the so-called asymmetric vehicle-following phenomenon. Siuhi [5] affirmed that different Δ*t*, *m*, and *k* values are necessary to also account for vehicle types of the leader and the follower.

Wang et al. studied interdriver and intradriver heterogeneities using vehicle trajectory data collected at the A2 Motorway in Utrecht, the Netherlands [7]. They calibrated the Helly model, Gipps model, and Intelligent Driver model. They found that, for the majority of the drivers, (i) the Δ*t* for deceleration was smaller than that for acceleration; (ii) when the same vehicle-following model was fitted to the data, the fitted parameter values for acceleration and deceleration conditions were different; and (iii) the best fitted model took different forms in acceleration and in deceleration.

Ossen and Hoogendoorn presented the results of five vehicle-following models which were calibrated against vehicle trajectory data collected at the A2 Motorway in Utrecht and the A15 Motorway in Rotterdam, the Netherlands [8]. They compared the models when a car was following a car and when a car was following a truck. Among the findings were (i) different vehicle-following models best fitted different passenger cars; (ii) truck tended to be driven in a relatively lower speed variance compared to passenger cars; and (iii) the desired headways are lower when a car was following a car compared to a car following a truck. Their findings showed interdriver heterogeneity between passenger cars and well as the heterogeneity depending on the leader's vehicle type.

The above recent studies have shown that heterogeneities in vehicle-following behavior exist (i) for the same follower during acceleration and deceleration; (ii) for the same follower, when the leaders are of different vehicle types; (iii) between different followers, even when the leader-follower pairs are of the same vehicle combination.

### 2.2. Self-Organizing Feature Map

The SOM, introduced by Kohonen [20], is motivated by the self-organization characteristics of the human cerebral cortex. The SOM can learn to detect regularities and correlations in its input with its existing memory and adapt its responses to that input accordingly.

**Figure 1:**
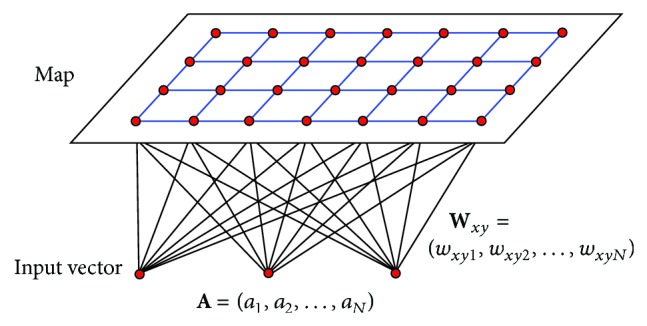
General architecture of self-organizing feature map.

The SOM is one type of neural networks [21]. The network topology and unsupervised training scheme make it different from the commonly known neural networks. A SOM is usually a two-dimensional grid, as shown in Figure 1. The map is usually square, but can be of any rectangular or hexagonal shape. Each point on the grid, denoted by its coordinate position (*x*, *y*), has a neuron and its associated weight vector **W**_*xy*_. The *N*-dimensional weight vector **W**_*xy*_ = (*w*_*xy*1_, *w*_*xy*2_,…, *w*_*xyn*_,…, *w*_*xyN*_) represents the centroid of a data cluster of similar training vectors. The weight vectors are collectively known as the SOM's memory.

The SOM is a mapping technique to project an *N*-dimensional input space to a two-dimensional space, effectively performing a compression of the input space. When an input vector **A** = (*a*_1_, *a*_2_,…, *a*_*n*_,…, *a*_*N*_) is presented to the SOM, the “distance” between **A** and each of the weight vectors in the entire SOM is computed. The neuron whose weight vector is “closest” to **A** will be declared as the “winner” and has its output set to 1, while others are set to 0. Mathematically, the output *b*_*xy*_ of a neuron located at (*x*, *y*) is

(2) $$\begin{array}{c} b_{xy}=\left\{\begin{array}{cc} 1, & \text{if}\;\;\left\|A-W_{xy}\right\|=\underset{\forall i,j}{\min}\left\{\left\|A-W_{ij}\right\|\right\},\\ 0, & \text{otherwise}, \end{array}\right. \end{array}$$

where ‖‖ represents the Euclidean distance and *i* and *j* are indices of the grid positions in the SOM. The input vectors that are categorized into the same cluster, that is, the same winning neuron, have the same output. In the above equation, as in most SOM applications, *b*_*xy*_ is coded as a binary variable. However, in some real world applications, it is possible for *b*_*xy*_ to be a discrete or continuous variable, as illustrated later in this paper.

The training of a SOM is to code all the **W**_*xy*_ so that each of them represents the center of a cluster of similar training vectors. Once trained, the **W**_*xy*_ is known as a prototype vector (of the cluster it represents). The SOM training is based on a competitive learning strategy. During training, the winning neuron, denoted by (*X*, *Y*), adjusts its existing weight vector **W**_*XY*_ towards the input vector **A**. Neurons that are neighboring to the winning neurons on the map also learn part of the features of **A**. For each neuron, the weight vector during training step *t* is updated as

(3) $$\begin{array}{c} W_{xy}^{T}\left(t+1\right)=W_{xy}^{T}\left(t\right)+h_{xy,XY}\left(t\right)\left\|A^{T}-W_{xy}^{T}\left(t\right)\right\|. \end{array}$$

The function *h*_*xy*,*XY*_(*t*) is the neighborhood function which embeds the learning rate. The value *h*_*xy*,*XY*_(*t*) decreases with increasing *d*_*xy*,*XY*_, the distance between the winning neuron at (*X*, *Y*) and the neuron of interest at (*x*, *y*). To achieve convergence, it is necessary that *h*_*xy*,*XY*_(*t*) → 0 as *t* → *∞*. More details on the SOM training may be found in [22].

In transportation engineering, the SOM has recently been applied to vehicle classification [23] and traffic data classification [23, 24], among others.

## 3. Data Professing

The data used in this research was part of the NGSIM vehicle trajectory data collected in the northbound direction of Interstate 80 Freeway at Emeryville, CA, on April 13, 2005 (Wednesday), from 4:00 p.m. to 4:15 p.m. [25]. The downloaded data consisted of the trajectories of individual vehicles at 0.1 second intervals as they traveled across the 503 m segment. There were six northbound lanes at this site. The leftmost lane (lane 1) was the High Occupancy Vehicle lane, while the two rightmost lanes (lanes 5 and 6) have many weaving or merging movements between an on-ramp and an off-ramp. To ensure that the data analyzed was mostly through movements, only data in lanes 2, 3, and 4 was extracted, processed, and analyzed. During this 15-minute period, traffic volume ranged from 1278 to 1414 vphpl, and the average space-mean-speed ranged from 27.9 to 30.1 km/h [25].

The data was filtered to meet the following criteria.

1. The followers must be passenger cars but the leaders could be passenger cars or trucks.
2. Each pair of leader and follower must have at least 5.0 seconds of interaction. If the required interaction time is too long, few pairs of vehicles could be extracted from the 503 m segment. However, vehicle pairs must have a few seconds of continuous interactions so as to observe the follower's acceleration or deceleration behavior. The 5.0 seconds was arbitrarily selected as a compromise between these two conflicting factors.
3. Gap at time *t* is defined as *x*_*l*_(*t*) − *x*_*f*_(*t*) − *L*_*l*_, where *L*_*l*_ is the length of the lead vehicle. This is because the following drivers usually judge the following distance by looking at the rear end of the lead vehicle and use the lead vehicle's brake lights to detect the leader's sudden deceleration. Vehicles following with a large gap behind the leaders are unlikely to have interaction with the leaders. Therefore, according to [26], the vehicle pairs with a maximum spacing below 50 m were more likely to be in vehicle-following situations, so only data with gap of 50 m of shorter were processed further.
4. The time lag (Δ*t*) for acceleration was assumed to be 0.80 second while that for deceleration was assumed to be 0.70 second. These values were taken from the average values reported by [5]. Although other studies (e.g., [1, 8, 15–18]) have reported different reaction times, the above average values used by [5] were adopted as they were derived from the NGSIM vehicle trajectory data collected at the closest available site (U.S. 101 Freeway in Los Angeles, CA) and then validated against the data collected at the Interstate 80 Freeway site at Emeryville, CA.
5. The vehicle velocities and accelerations were estimated according to the recommendations of [26]. At every 0.1 second intervals, a vehicle's instantaneous velocity was calculated from the longitudinal difference in the coordinates “Local *Y*”. The velocity was further “smoothed” by taking the average value within the past 0.5 second intervals. At any time instant *t*, *x*_*l*_(*t*) and *x*_*f*_(*t*) were the vehicle positions at *t*, ${\dot{x}}_{l}\left(t\right)$ and ${\dot{x}}_{f}\left(t\right)$ were the average velocities from *t* − 0.4 to *t* seconds, while ${\ddot{x}}_{f}(t+\Delta t)$ was the follower's acceleration or deceleration from *t* + Δ*t* − 0.4 to *t* + Δ*t* seconds. This ${\ddot{x}}_{f}(t+\Delta t)$ value was computed from $\left({\dot{x}}_{f}(t+\Delta t-0.4)-{\dot{x}}_{f}(t+\Delta t)\right)/0.5$.
6. The data were then checked for possible errors. For example, *x*_*l*_(*t*) − *x*_*f*_(*t*) − *L*_*l*_ must be greater than 0 m, and ${\dot{x}}_{l}\left(t\right)$ and ${\dot{x}}_{f}\left(t\right)$ must be between 0 and 22 m/s (80 km/h). It was discovered that 381 out of 106,644 vectors did not meet the abovementioned filtering criteria, including gap ≤50 m. These 381 vectors were discarded.

The processed data consisted of 1,347 pairs of “car following car” and 66 pairs of “car following truck” scenarios. Data from 897 randomly selected pairs of “car following car” were assembled as the training data set. The other 450 pairs of “car following car” formed test data set I. Since 66 pairs of “car following truck” were insufficient to form a training data set, they were assembled to form test data set II. The training data set had 67,778 vectors (at 0.5 second intervals). The test data set I had 33,803 vectors while test data set II had 4,675 vectors. Each vector (at time *t*) had four components: ${\ddot{x}}_{f}(t+\Delta t)$, ${\dot{x}}_{f}\left(t\right)$, ${\dot{x}}_{l}\left(t\right)-{\dot{x}}_{f}\left(t\right)$, *x*_*l*_(*t*) − *x*_*f*_(*t*) − *L*_*l*_. The minimum and maximum values of each component are shown in Table 1. The accelerations were found to be between −3.41 and 3.41 m/s^2^ which were within the values used in the design of stopping sight distance [26]. Note that, unlike formula (1), the follower's velocity ${\dot{x}}_{f}(t)$ has no time lag. This was deliberately set so that our model input was consistent with most of the vehicle-following models, including the one used in [5, 6].

## 4. Training of Self-Organizing Feature Map

### 4.1. Architecture and Mapping Framework

**Figure 2:**
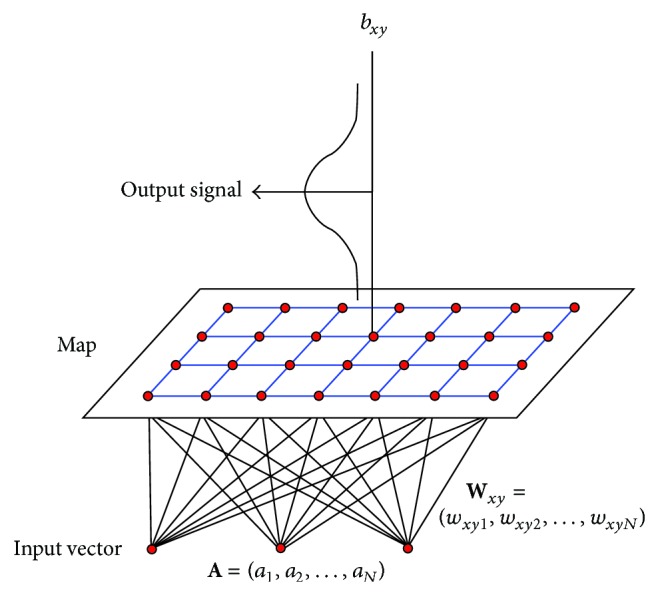
Architecture of self-organizing feature map for vehicle-following.

The concept of this research was to first construct a SOM with weight vectors that represent the prototype vehicle-following stimuli for the “car following car” scenarios. The acceleration response of each training vector was then associated with the winning neuron. With the numerous training vectors, it was possible to plot and analyze the distribution of acceleration response associated with each neuron in the SOM (see the distribution of *b*_*xy*_ in Figure 2). Furthermore, the trained SOM was used to classify the vehicle-following stimuli embedded in the input vectors in the test data sets. Once the winning neuron had been identified, statistical parameters of the response of the winning neuron could be used to study the heterogeneous behavior in vehicle-following.

As the input and weight vectors represented the vehicle-following stimulus, the follower's velocity, relative velocity, and gap, following components were selected to form the input vectors. That is, $A=({\dot{x}}_{f}(t),{\dot{x}}_{l}(t)-{\dot{x}}_{f}(t),x_{l}(t)-x_{f}(t)-L_{l})$. These three components were selected because they are commonly found in vehicle-following models, such as the GHR, Helly, and Gipps models.

### 4.2. Training

The training of the SOM was performed with the MATLAB Neural Network Toolbox [27] in a standard desktop computer. Before the SOM training, each component of the input vector was linearly scaled to [0,1] between its minimum and maximum values in the data set, that is, *a*_*n*_ ∈ [0,1], *n* = 1,2, 3. The training was conducted over two phases: ordering and tuning. In the ordering phase, the weight vectors were adjusted at relatively larger magnitudes. The initial neighborhood radius was arbitrarily set to 3.0, learning rate set to begin at 0.15, and the number of steps set to 1000. The neighborhood size started at an initial distance and decreased as training proceeded. During the tuning phase, only weights of the winning neuron and its immediate neighbors were updated at relatively smaller magnitudes. During this phase, the neighborhood distance was fixed at 1.0, learning rate was fixed at 0.02, and the number of tuning steps was 100.

The size of the SOM was selected in consideration of the following two factors. First, the grid has to be large enough so that there were sufficient neurons to distinguish the varied stimuli among the prototype weight vectors. Since the SOM has three input components and the value of each component may be viewed at five levels (e.g., ${\dot{x}}_{f}\left(t\right)$ may be described as very slow, slow, moderate, fast, or very fast), there would be 125 possible combinations of input levels. Second, the number of neurons must be small enough such that most, if not all neurons have sufficient winning frequencies (sample sizes) to observe the distribution of the response values. This was especially critical for test data set II which had relatively fewer pairs of “car following truck” observations. After some initial trials which involved SOMs with different number of neurons and with different arrangements (square grid, rectangular grid and linear) in the map, the SOM was determined to have 121 neurons arranged in an 11 × 11 square grid. Although the 121 neurons were fewer than the 125 suggested earlier, it could be used as some combinations of ${\dot{x}}_{f}\left(t\right)$, ${\dot{x}}_{l}\left(t\right)-{\dot{x}}_{f}\left(t\right)$, *x*_*l*_(*t*) − *x*_*f*_(*t*) − *L*_*l*_ values were not possible in practical vehicle-following situations.

## 5. Results and Discussions

### 5.1. Distribution of Stimulus

**Figure 3:**
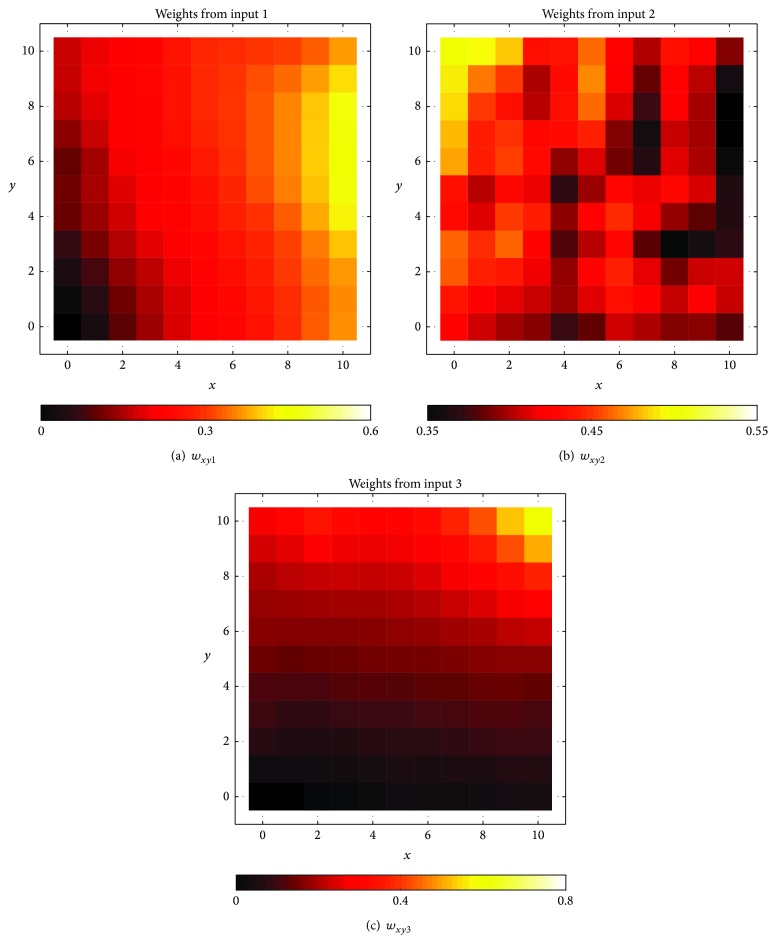
Maps of weight components after SOM training.

Figure 3 plots the two-dimensional maps of the three weight components of the trained SOM. The neurons are numbered according to the (*x*, *y*) coordinates in the grid, where *x* = 0,1,…, 10 and *y* = 0,1,…, 10. The darker colors represent smaller weight values while the lighter colors represent higher weight values. Because *a*_*n*_ ∈ [0,1], *n* = 1,2, 3 and because of (3), *w*_*xyn*_ ∈ [0,1], *n* = 1,2, 3. Note that the ranges of *w*_*xy*1_, *w*_*xy*2_, and *w*_*xy*3_ values are different. This is because the extreme weight values in the training vectors did not occur often, and formula (3) will update the weights to the normally encountered ranges. The statistics of the weight values are summarized in Table 2. The three maps in Figure 3 show that, after training, the values of *w*_*xy*1_, *w*_*xy*2_, and *w*_*xy*3_ change gradually from one corner to the opposite corner in their respective maps. This indicates that the weight values (within their respective ranges) have been distributed spatially among the prototype vectors, with the neighboring vectors having similar weights.

From the maps in Figure 3, it can be seen that the neurons at the lower left corner has low follower's velocities, almost zero relative velocities (*w*_*xy*2_ value in the mid-range) and small gaps. They represent the state where vehicles are queuing in congested conditions. In this condition, the follower is expected to accelerate or decelerate with small magnitudes. The neurons located at the top right corner of the grid represent stimulus with relatively high follower's velocities, negative relative velocities (*w*_*xy*2_ less than midvalue), and large gaps. This condition indicates that the follower is closing in to the leader from a distance (but may not necessarily decelerate). The neurons at the top left corner have moderate follower's velocities, high relative velocities, and moderate gaps. They represent the scenario that the lead vehicle is accelerating away from the follower. The follower may then respond by accelerating. The neurons at the bottom right corner have weight vectors that have moderately high follower's velocities, negative relative velocities, and small gaps. These prototype vectors represent the condition that the follower is quickly closing in to the leader. The driver of the following vehicle is likely to apply his/her brake.

### 5.2. Distribution of Mean Response

**Figure 4:**
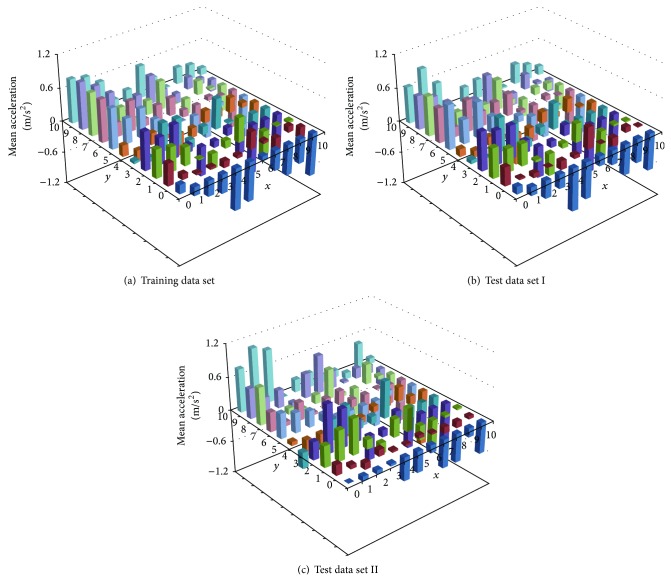
Maps of average acceleration.

For each neuron, the mean response (average follower's acceleration) computed from the winning vectors is next plotted in Figure 4.  Figure 4(a) shows the distribution of mean response calculated from the training data set. For each *x* value in the map, as *y* increases from 0 to 10, the mean response changes from deceleration to acceleration. For each *y* value in the map, as *x* increases from 0 to 10, the mean response changes from acceleration to deceleration. The maximum acceleration occurs near *x* = 0, *y* = 10, which is the top left corner of the SOM as shown in Figure 3. On the other hand, the maximum deceleration occurs near *x* = 10, *y* = 0, which is the bottom right corner of the SOM in Figure 3.

The distributions of mean response among the vectors in the two test data sets are presented in Figures 4(b) and 4(c), respectively. These figures exhibit similar patterns, indicating that the weight vectors had converged towards the end of the SOM training. Thus, viewed in conjunction with Figure 3, it can be concluded that the SOM has learned to capture the prototype characteristics of most of the vehicle-following stimuli among the training data.

The mean and variance of response associated with each neuron were next analyzed. The minimum variance of acceleration occurred at neuron (*x* = 0, *y* = 0). At this neuron, the variance is 0.41 m^2^/s^4^ or standard deviation of 0.64 m/s^2^. The variances of the other neurons all exceeded this magnitude. Another way to view the high variability of the acceleration is by means of coefficient of variation, which is the ratio of standard deviation over mean. All the absolute values of coefficients of variation exceeded 1.19, indicating high variability in acceleration response.

**Figure 5:**
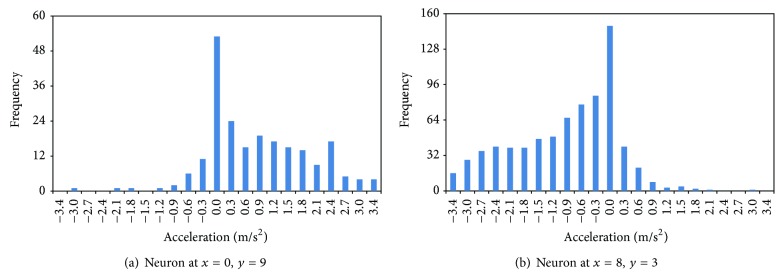
Distribution of response for the same stimulus categories.

Figure 5 plots the distribution of follower's acceleration for input vectors (in the training data set) that had winning neurons at (*x* = 0, *y* = 9) and (*x* = 8, *y* = 3), respectively. The neuron at (*x* = 0, *y* = 9), as reflected in Figure 3, has moderate follower's velocity, high relative velocity, and moderate gap. In such a condition, most of the followers are expected to respond with acceleration. The accelerations as shown in Figure 5(a) were distributed between [−3.04,3.41] m/s^2^ with a mean of 0.85 m/s^2^. The neuron at (*x* = 8, *y* = 3) belongs to the input state that has high follower's velocities, negative relative velocities, and small gaps. Majority of the drivers facing this situation will decelerate to avoid a rear-end collision. As shown in Figure 5(b), the response ranges from [−3.41,2.97] m/s^2^ with the mean of −0.94 m/s^2^. Moreover, for both neurons, the modes occurred at 0 m/s^2^. This is because the followers may choose not to act at the present time step; they may have responded at an earlier or later time step.

The analysis in this subsection and Figure 5 has shown that, given similar stimuli (input vectors that have the same winning neuron), the follower's response is not deterministic. The variation in the response may be due to the driving behavior between drivers (interdriver heterogeneity), the inconsistency of the same driver (intradriver heterogeneity), or when the leaders belong to different types of vehicle (inter-vehicle-type heterogeneity). Note that the term interdriver heterogeneity also implicitly includes the varied acceleration response caused by the different performance characteristics of the same type of vehicle (e.g., cars). These three types of heterogeneities will be demonstrated in the next three subsections.

### 5.3. Interdriver Heterogeneity

**Figure 6:**
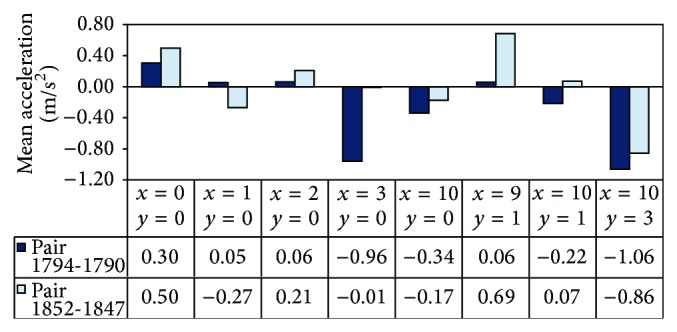
Differences in mean response between two followers.

To demonstrate interdriver heterogeneity, data from two pairs of passenger cars in test data set I was fed into the trained SOM and the distributions of their responses were compared. Due to limitations on space, we chose two pairs which share the most number of the same winning neurons to demonstrate the interdriver heterogeneity. The first pair was denoted as Pair 1794-1790, in which the follower's Vehicle Identification Number (VIN) in the NGSIM data set was 1794 and the leader's VIN was 1790. The second pair was Pair 1852-1847. For each pair of cars, the vehicle trajectories for at least 68 continuous seconds were extracted, resulting in more than 136 vectors at 0.5 second intervals. During the observed duration, the vehicle-following stimuli of Pair 1794-1790 were spread over 30 winning neurons. The corresponding number of winning neurons for Pair 1852-1847 was 23.  Figure 6 represents these two followers' mean acceleration responses associated with the eight common winning neurons. As shown in the figure, the two followers (VINs 1794 and 1852) had different mean acceleration magnitudes for the same winning neuron. In two of the winning neurons, the signs of the accelerations are opposite. Overall, VIN 1794 has higher magnitudes of acceleration while VIN 1852 has heavier deceleration. The differences may be caused by the followers' driving habits.

### 5.4. Intradriver Heterogeneity

**Figure 7:**
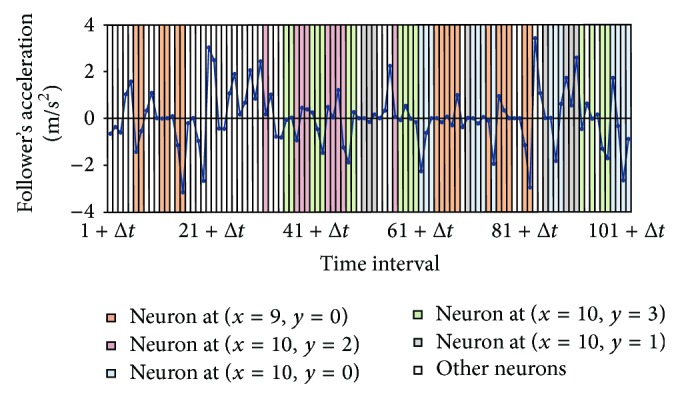
Acceleration profile of selected vehicle pair and winning neurons.

Another pair of passenger cars (Pair 350-346) in test data set I was selected to illustrate that, even if the same driver is presented with similar stimuli, his/her response may be inconsistent. This pair of vehicles has 50.5 seconds of vehicle-following observations, resulting in 101 vectors at 0.5 second intervals.  Figure 7 plots the follower's acceleration profiles over the duration of observation. The vertical color coded bars represent the winning neurons identified by the SOM. The Δ*t* in *t* + Δ*t* in the horizontal axis is to account for the time lag when the stimulus occurs at time *t*. Five neurons are highlighted here as they have sufficient winning frequencies for subsequent analysis.

**Figure 8:**
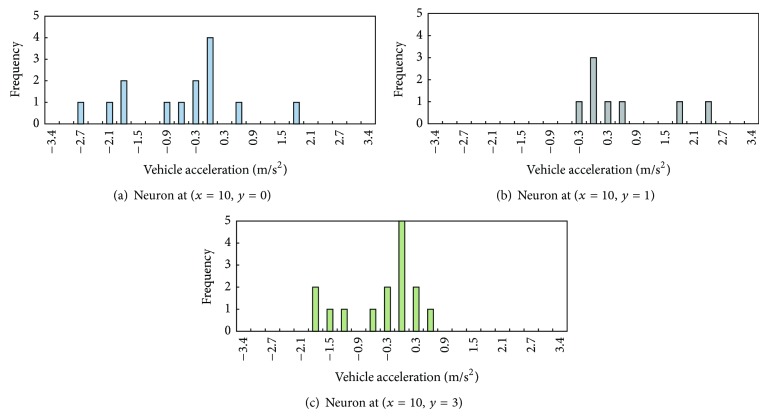
Distribution of response by VIN 350.

Figure 8 shows the distributions of VIN 350's responses in three of the five winning neurons identified in Figure 7. According to Figure 4, on average, drivers decelerate in neurons (*x* = 10, *y* = 0), (*x* = 10, *y* = 1), and (*x* = 10, *y* = 3). It appears that, on average, VIN 350 has the same deceleration signs at neurons (*x* = 10, *y* = 0) and (*x* = 10, *y* = 3) which is consistent with the driver population in the training and test data sets. However, the driver of VIN 350 has, on average, acceleration response at neuron (*x* = 10, *y* = 1) (see Figure 8(b)) while the average response in the data sets is deceleration. As plotted in Figure 8, when faced with similar inputs belonging to the same winning neuron, the driver of VIN 350 had varied responses. This evidence suggests that the same driver responded inconsistently when the stimulating factors are considered analogous.

### 5.5. Inter-Vehicle-Type Heterogeneity

In this subsection, the distribution of responses among the vectors in test data sets I and II was compared. Test data set I consisted of data from “car following car” scenarios while test data set II consisted of “car following truck” scenarios. For each stimulus at neuron (*x*, *y*), a two-tail paired *t*-test was conducted to see if the difference between the mean responses is significant. Of the 121 paired *t*-tests conducted, the results in eight neurons listed in Table 3 show significant differences between the two means at 0.05 level of significance (*P* value less than 0.05). This suggests that the follower's response is dependent on the vehicle type of the leader. At the other 113 neurons, the paired *t*-test showed no significant difference between the two means. The reason is most likely due to the high variances in the acceleration of the followers (i.e., inter- and intradiver heterogeneity).

## 6. Conclusions, Limitations, and Potential Research Directions

This paper has applied the SOM as a nonparametric approach in modeling vehicle-following behavior. Vehicle trajectory data, when both leaders and followers were passenger cars, was used to train a SOM with 121 neurons arranged in a 11 × 11 grid. The follower's velocity, relative velocity, and gap were the components of the weight vectors. After training, the SOM represented the vehicle-following stimuli among its weight vectors.

Selected pairs of vehicle trajectory data were fed into the trained SOM. The SOM identified similar stimuli between the different followers so that the acceleration responses could be compared. The results revealed that with similar stimuli (i) heterogeneity exists between different car drivers when following cars; (ii) heterogeneity exists for a car driver when following the same car; and (iii) heterogeneity exists for car drivers when the leaders belong to different vehicle types (car versus trucks).

One of the advantages of the SOM (compared to conventional vehicle-following models) is its ability to map the essential stimulus components with the acceleration response without having users specify the function form of the vehicle-following equation or perform parameter calibration. Although this research focused on the construction of a SOM based on “car following car” scenario, it is possible to construct other SOMs each tailored to a specific combination of vehicle types between the leader and follower, such as “car following truck,” “truck following car,” or “truck following truck.” One may also need to construct several sets of SOMs, with each set for a different driving context, for example, highways versus urban arterials.

The SOM also has a potential to replace the conventional vehicle-following models currently being used in microscopic traffic simulation tools. To apply a trained SOM for this purpose, a user needs to compare the vehicle-following stimulus components with the prototype vectors to locate the winning neuron at (*X*, *Y*). The follower's response is then taken from the probability distribution of *b*_*XY*_. The acceleration response is thus stochastic. It is very likely that the acceleration is further subjected to some rules to prevent sudden fluctuation from one interval to the next. This is beyond the scope of this paper and is a subject of future research.

Although this research has explored the use of SOM to model vehicle-following and used it to study heterogeneities in the follower's behavior, there are several limitations due to assumptions in this research. These limitations have suggested possible directions of future research. First, the SOM was developed from data gathered in the afternoon peak period. The SOM's prototype vectors may not fully cover the entire input space during the off-peak traffic conditions. Second, the SOM was trained with data from one freeway site. It would be interesting to test the transferability of the SOM to other sites. Third, fixed reaction times had been used in the processing of data. It is known that reaction times vary for the same driver and between drivers. That is, reaction time contributes to heterogeneities. However, without assuming fixed reaction times, it was very difficult, if not impossible to proceed with the analyses presented in this paper. Future research should explore a new methodology to estimate reaction time or incorporate reaction time into the SOM's input or output. Fourth, during training, the number of neurons and the neighborhood radius of SOM are two crucial parameters affecting SOM's clustering performance. The paper determined these parameters empirically based on the size of data set and the operation speed of computers. Analytical methods need to be further developed to give a remark regarding how to determine these parameters in a more reasonable manner. Fifth, this research had manually inspected the weight distributions among the neurons (Figure 3) to ascertain the convergence of weights at the end of SOM training. An objective method of assessing the weight convergence would be helpful in future SOM applications.

## Figures and Tables

**Table 1:** Minimum and maximum values of the components in the training and test vectors.

Date set	${\ddot{x}}_{f}\left(t+\Delta t\right)$ (m/s^2^)		${\dot{x}}_{f}\left(t\right)$ (m/s)		${\dot{x}}_{l}\left(t\right)-{\dot{x}}_{f}\left(t\right)$ (m/s)		*x*_*l*_(*t*) − *x*_*f*_(*t*) − *L*_*l*_ (m)	
	min	max	min	max	min	max	min	Max
Training set	−3.41	3.41	0	20.20	−12.24	14.38	0.03	49.98
Test set I	−3.41	3.41	0	21.30	−13.41	16.68	0.01	49.86
Test set II	−3.41	3.41	0	14.84	−7.90	7.61	0.03	48.67

**Table 2:** Statistics of the weight values of the trained SOM.

	*w*_*xy*1_ Flower's velocity	*w*_*xy*2_ Relative velocity	*w*_*xy*3_ Gap
Number of prototype vectors	121	121	121
Average	0.3202	0.4492	0.2692
Standard deviation	0.1088	0.0259	0.1282
Minimum	0.0407	0.3956	0.0770
Maximum	0.5349	0.4152	0.7373

**Table 3:** Two-tail *t*-tests for inter-vehicle-type heterogeneity.

Neuron	Leader's vehicle type	Sample size	Mean (m/s^2^)	Variance (m^2^/s^4^)	*t*-stat	*P* value
*x* = 4 *y* = 0	Car	324	−0.78	1.47	−2.080	0.038
	Truck	52	−0.41	−0.41		

*x* = 7 *y* = 2	Car	283	0.00	0.76	2.133	0.034
	Truck	50	−0.27	−0.27		

*x* = 8 *y* = 4	Car	293	−0.21	0.84	2.158	0.032
	Truck	35	−0.56	−0.56		

*x* = 0 *y* = 6	Car	254	0.79	1.05	2.524	0.012
	Truck	65	0.45	0.45		

*x* = 5 *y* = 6	Car	185	0.11	0.72	2.098	0.037
	Truck	40	−0.21	−0.21		

*x* = 1 *y* = 8	Car	94	0.28	0.74	2.195	0.030
	Truck	46	−0.05	−0.05		

*x* = 2 *y* = 10	Car	311	0.53	0.98	−1.977	0.049
	Truck	26	0.94	0.94		

*x* = 8 *y* = 10	Car	383	0.37	0.93	2.900	0.004
	Truck	36	−0.11	−0.11		

## References

[1] Brackstone M., McDonald M. (1999). Car-following: a historical review. *Transportation Research F: Traffic Psychology and Behaviour*.

[2] Panwai S., Dia H. (2005). Comparative evaluation of microscopic car-following behavior. *IEEE Transactions on Intelligent Transportation Systems*.

[3] Gazis D. C., Herman R., Rothery R. W. (1961). Nonlinear follow-the-leader models of traffic flow. *Operations Research*.

[4] Gazis D. C. (2002). *Traffic Theory*.

[5] Siuhi S. (2009). *Parametric study of stimulus-response behavior incorporating vehicle heterogeneity in car-following models [Ph.D. dissertation]*.

[6] Siuhi S., Kaseko M. Parametric study of stimulus-response behavior for car-following models.

[7] Wang H., Wang W., Chen J., Jing M. (2010). Using trajectory data to analyze intradriver heterogeneity in car-following. *Transportation Research Record*.

[8] Ossen S., Hoogendoorn S. P. (2011). Heterogeneity in car-following behavior: theory and empirics. *Transportation Research. Part C: Emerging Technologies*.

[9] Helly W., Herman R. Simulation of bottlenecks in single lane traffic flow.

[10] Gipps P. G. (1981). A behavioural car-following model for computer simulation. *Transportation Research B*.

[11] Treiber M., Hennecke A., Helbing D. (2000). Congested traffic states in empirical observations and microscopic simulations. *Physical Review E*.

[12] ITT (2001). x 5.0. *Contract no.*.

[13] PTV (2009). *VISSIM Version 5.20 User Manual*.

[14] Brockfeld E., Kühne R. D., Wagner P. (2004). Calibration and validation of microscopic traffic flow models. *Transportation Research Record*.

[15] Ranjitkar P., Nakatsuji T., Asano M. (2004). Performance evaluation of microscopic traffic flow models with test track data. *Transportation Research Record*.

[16] Ossen S., Hoogendoorn S. P. (2005). Car-following behavior analysis from microscopic trajectory data. *Transportation Research Record*.

[17] Punzo V., Simonelli F. (2005). Analysis and comparison of microscopic traffic flow models with real traffic microscopic data. *Transportation Research Record*.

[18] Ossen S., Hoogendoorn S. P., Gorte B. G. H. (2006). Interdriver differences in car-following a vehicle trajectory-based study. *Transportation Research Record*.

[19] Kesting A., Treiber M. (2008). Calibrating car-following models by using trajectory data: methodological study. *Transportation Research Record: Journal of the Transportation Research Board*.

[20] Kohonen T. (1987). *Self-Organization and Associative Memory*.

[21] Haykin S. (1998). *Neural Networks: A Comprehensive Foundation*.

[22] Engelbrecht A. P. (2002). *Computational Intelligence*.

[23] Sun C., Ritchie S. G., Oh S. (2003). Inductive classifying artificial network for vehicle type categorization. *Computer-Aided Civil and Infrastructure Engineering*.

[24] Chen Y., Zhang Y., Hu J., Yao D. Pattern discovering of regional traffic status with self-organizing maps.

[25] Cambridge (2005). *NGSIM Data Analysis Report (4:00 p.m. to 4:15 p.m.) Summary Report*.

[26] Punzo V., Borzacchiello M. T., Ciuffo B. (2011). On the assessment of vehicle trajectory data accuracy and application to the Next Generation SIMulation (NGSIM) program data. *Transportation Research Part C: Emerging Technologies*.

[27] AASHTO (2004). *A Policy on Geometric Design of Highways and Streets*.

